# DMFF-Net: A dual encoding multiscale feature fusion network for ovarian tumor segmentation

**DOI:** 10.3389/fpubh.2022.1054177

**Published:** 2023-01-11

**Authors:** Min Wang, Gaoxi Zhou, Xun Wang, Lei Wang, Zhichao Wu

**Affiliations:** ^1^School of Life Sciences, Tiangong University, Tianjin, China; ^2^School of Control Science and Engineering, Tiangong University, Tianjin, China; ^3^College of Computer Science and Technology, China University of Petroleum, Qingdao, China; ^4^Department of Gynecology, The Affiliated Hospital of Qingdao University, Qingdao, China

**Keywords:** ovarian cancer, dual encoding, residual block, single dense aggregation block, multiscale feature fusion

## Abstract

Ovarian cancer is a serious threat to the female reproductive system. Precise segmentation of the tumor area helps the doctors to further diagnose the disease. Automatic segmentation techniques for abstracting high-quality features from images through autonomous learning of model have become a hot research topic nowadays. However, the existing methods still have the problem of poor segmentation of ovarian tumor details. To cope with this problem, a dual encoding based multiscale feature fusion network (DMFF-Net) is proposed for ovarian tumor segmentation. Firstly, a dual encoding method is proposed to extract diverse features. These two encoding paths are composed of residual blocks and single dense aggregation blocks, respectively. Secondly, a multiscale feature fusion block is proposed to generate more advanced features. This block constructs feature fusion between two encoding paths to alleviate the feature loss during deep extraction and further increase the information content of the features. Finally, coordinate attention is added to the decoding stage after the feature concatenation, which enables the decoding stage to capture the valid information accurately. The test results show that the proposed method outperforms existing medical image segmentation algorithms for segmenting lesion details. Moreover, the proposed method also performs well in two other segmentation tasks.

## 1. Introduction

**Figure 1:**
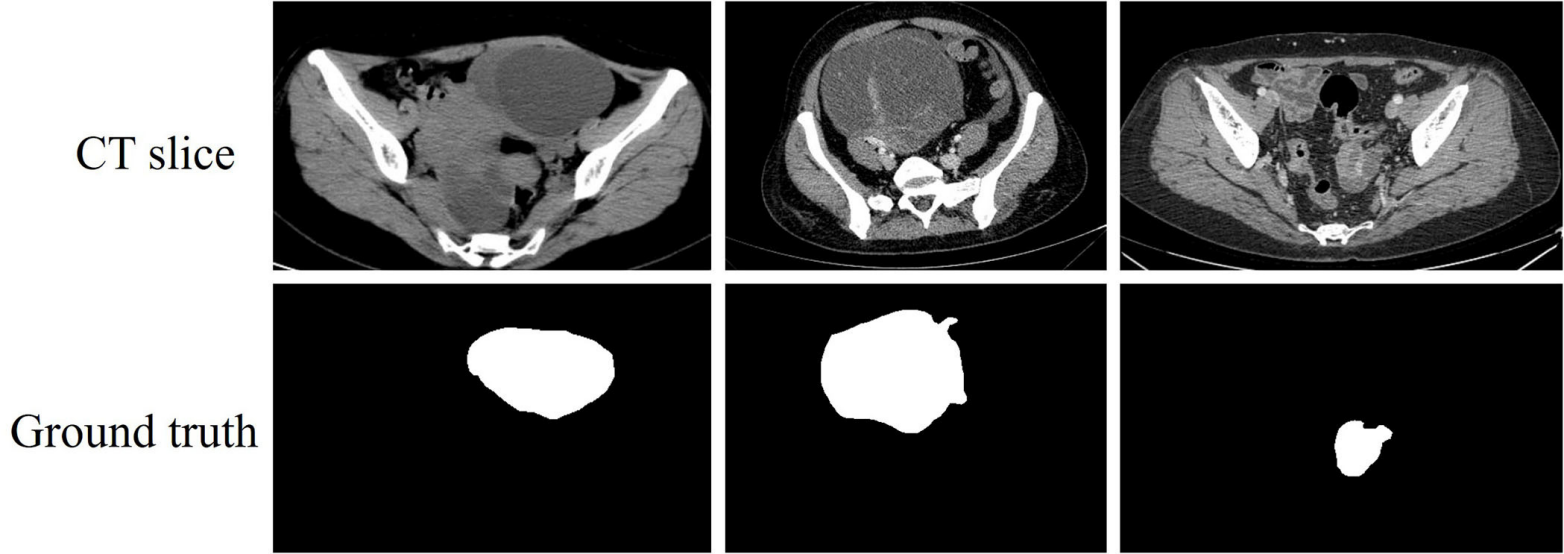
CT slices of ovarian tumor and their corresponding ground truth.

Ovarian cancer is one of the malignant tumors that the medical field has been devoted to treatment. Some data shows that the incidence of ovarian cancer ranks third below cervical cancer and endometrial cancer. However, the mortality rate of ovarian cancer is higher than both of them and ranks first among gynecological cancers, which is the biggest hidden threat to women's life and health (1). Accurate and rapid localization of the tumor area is beneficial for the diagnosis of ovarian cancer. Medical imaging technologies have matured in recent years, many imaging methods can visualize the lesion area and help the physicians diagnose the disease (2–4). Computed tomography (CT) is a method commonly applied to visualize ovarian tumor lesions, and its visualized slices are shown in Figure 1. The proliferation of CT data has led to a surge in physician review of CT images. The long and intense review work inevitably leads to misdiagnosis or missed diagnosis, so there is an urgent need for a computer aided diagnosis (CAD) technique to assist physicians in accurately outlining the lesion area.

Semi-automatic segmentation method is an early technique used to assist physicians in segmenting lesions. Sarty et al. (5) proposed a semi-automatic follicle segmentation method based on prior knowledge. The method first determines the approximate inner follicle wall boundary by a combination of interactive adjustment and computer detection, and then utilizes this boundary as the prior knowledge to automatically find the outer follicle wall boundary. The semi-automatic segmentation method alleviates some of the physician's work, but it still requires someone with professional knowledge to operate and is more expensive to use. Subsequently, some automatic segmentation methods no longer require prior knowledge, and Nawgaje and Kanphade (6) introduced a genetic algorithm for choosing the optimal threshold of image segmentation. The crossover probability and mutation probability of this algorithm are adjusted by the variance of the target and background.

The deep learning-based segmentation methods can better deal with the diversified images and the noise in the images, and have better robustness compared to the traditional methods (7–13). FCN proposed by Long et al. (14) has pioneered the development of encode-decode structure by replacing the fully connected layer at the end with a convolutional layer to obtain the class information of each pixel. Ronneberger et al. (15) proposed a symmetric U-shaped structure inspired by FCN specifically for segmentation of medical images. This method fuses features from the encoding stage relatively independently to the decoding stage, so that features can be utilized more completely. This design improves the uncertain segmentation of details by FCN, and many models have continued this structure thereafter. Li et al. (16) designed a new composite model. This model incorporates a spatial recurrent network into a simple U-Net for segmenting ovary and follicles. Wang et al. (17) proposed an improved U-shaped network. This model replaces the ordinary convolution with a convolutional layer combined with recursive and residual blocks, and embeds an attention mechanism in the skip connection.

The simple replacement of the sub-structure of network has limited improvement on the network performance, and more ideas are being developed. Many studies have shown that shallow features contain more positional and detailed information but low semantics; deep features have more semantic information but lack detailed features; different sizes of convolution kernels can obtain features of various receptive fields. Therefore, some methods have emerged to study multiscale feature fusion to improve segmentation performance. Xia et al. (18) addressed a multiscale dilated convolution model. The model utilizes different sizes of dilated convolution to form a feature pyramid to extract semantic information, which integrates features at different scales. Zheng et al. (19) designed a two-channel separated convolution module with residual connections in the coding layer. This module fuses the input image with the feature maps after two-channel separation convolution for multiscale feature fusion. Hu et al. (20) addressed a hybrid encoding structure. The method feeds the preprocessed images into a hybrid attention mechanism and a densely connected convolutional network to extract features, respectively. Then, the outputs of the two encoding paths are fused at the terminal to produce multiscale features. Shareef et al. (21) introduced a segmentation network containing multiple encoding paths. The network uses convolution kernels of different sizes to obtain multiscale features, and then the multiscale features of each level are fused to the corresponding decoding blocks by skip connections.

The encoding stage is the main process of feature extraction. The above multi-coding methods all perform the fusion of multiscale features at the end of the encoding path or between the encoding path and the decoding path, and there is no communication between the encoding paths to share feature information. This results in insufficient multiscale feature fusion in the encoding stage, which affects the final segmentation effect. In order to remedy the deficiencies of the above works, we propose a dual encoding multiscale feature fusion network (DMFF-Net) for ovarian tumor segmentation. Firstly, a multi-resolution 2D image input is proposed to reduce the network training burden and retain the detailed information of the high-resolution image. High-resolution images are input to a path consisting of single dense aggregation blocks, and low-resolution images are input to a path consisting of residual blocks. Secondly, a multiscale feature fusion block is proposed to perform feature exchange between two encoding paths. The multiscale features extracted by different modules can be fused with each other to enrich feature information. Finally, in order to highlight the effective information of the feature maps in the decoding stage, coordinate attention (22) is used after the feature concatenation.

## 2. Materials and methods

**Figure 2:**
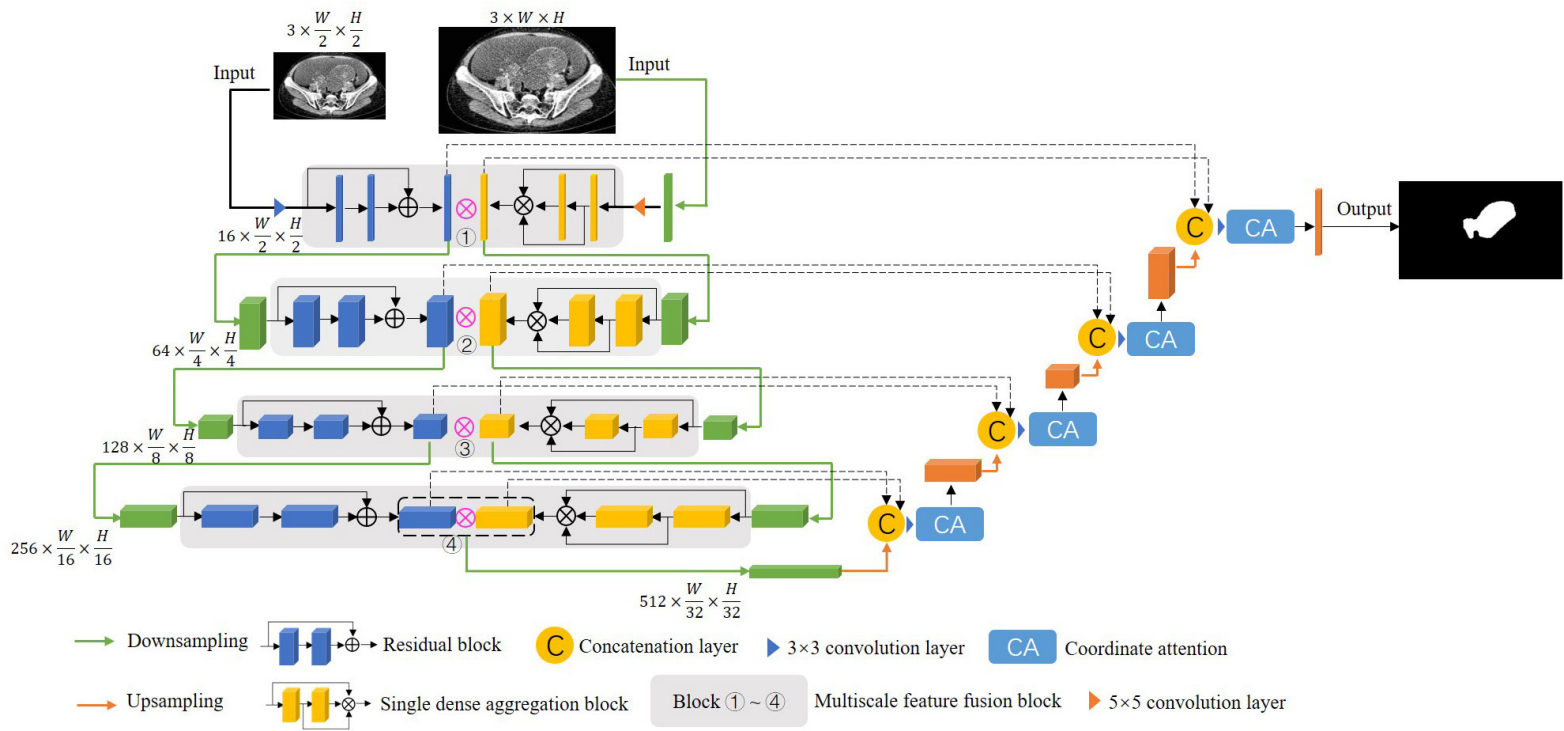
The structure of the proposed DMFF-Net in this paper.

In this paper, a multiscale feature fusion network with two different encoding paths is proposed, and the model enhances the communication of features in the encoding stage to make it superior in detail segmentation, and the proposed model is shown in Figure 2. The proposed model consists of the following main components: residual block, single dense aggregation block, multiscale feature fusion block and coordinate attention.

### 2.1. Dataset

Our dataset is 377 CT scans of ovarian tumor provided by the affiliated hospital of Qingdao University, and all data are desensitized and do not contain patients' personal information. The data involved in training and testing are randomly selected from this dataset, of which 80% is used as the training set and 20% as the testing set. In addition, a 5-fold cross-validation (23, 24) is performed on the training set with a 4:1 ratio of data used for training and validation, and the best model is tested in an unseen test set to obtain the final results, with all models involved in the comparison undergoing the above process. We extended the training data with rotation, Gaussian noise and mixup.

### 2.2. Data preprocessing

The size of the original CT images is 800 × 600, and we cropped the CT images with the ovarian tissue as the center to remove part of the black background which is irrelevant to the training.

**Figure 3:**
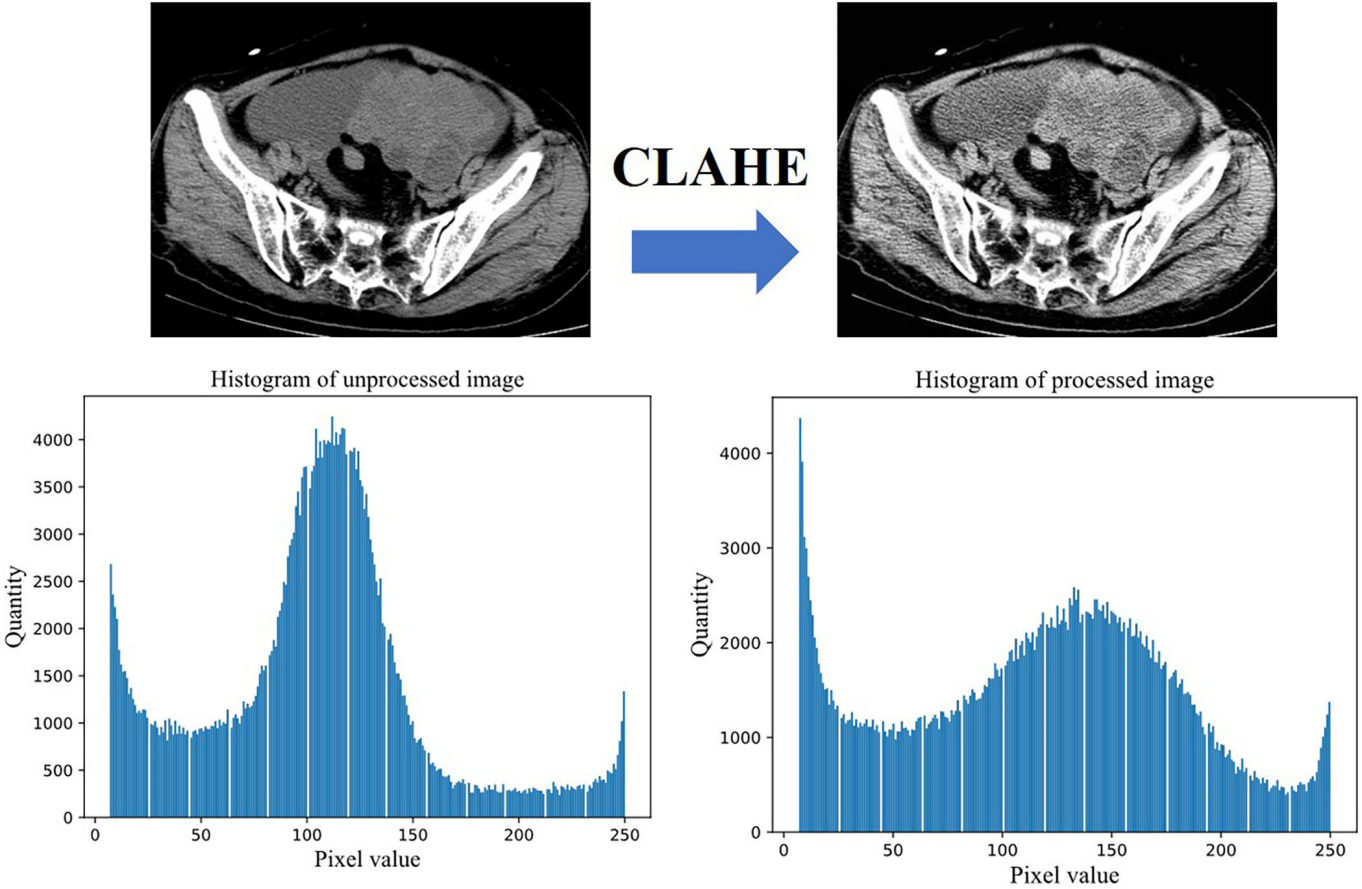
Effect and pixel histogram before and after image enhancement.

**Figure 4:**
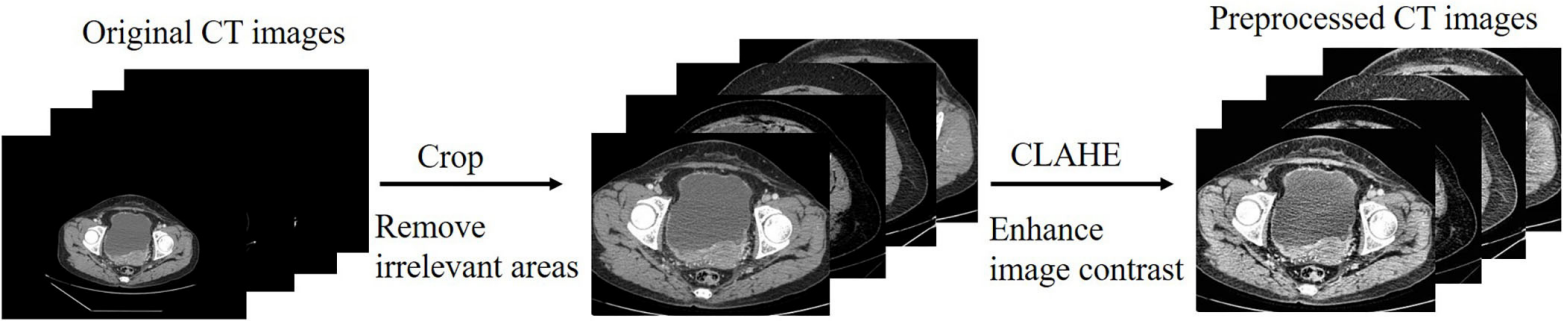
The process of CT image preprocessing.

The CT images are limited by the imaging principle, which leads to an unreasonable distribution of grayscale values in the generated images. We filtered the effect of black background and some extreme colors on the pixel histogram during the statistics, the comparison of the enhanced image with the original image is shown in Figure 3. We can see by the histogram of unprocessed image that the pixel values are too concentrated in some areas, and this causes a low contrast between the lesion and non-lesion areas of the ovarian tumor CT images we used. We enhanced the images using contrast limited adaptive histogram equalization (CLAHE), which is achieved by limiting the degree of contrast enhancement of the adaptive histogram. The histogram distribution of image pixels is more balanced and the image contrast is stronger after enhancement by CLAHE algorithm. The process of CT image preprocessing is shown in Figure 4.

### 2.3. Residual block

**Figure 5:**
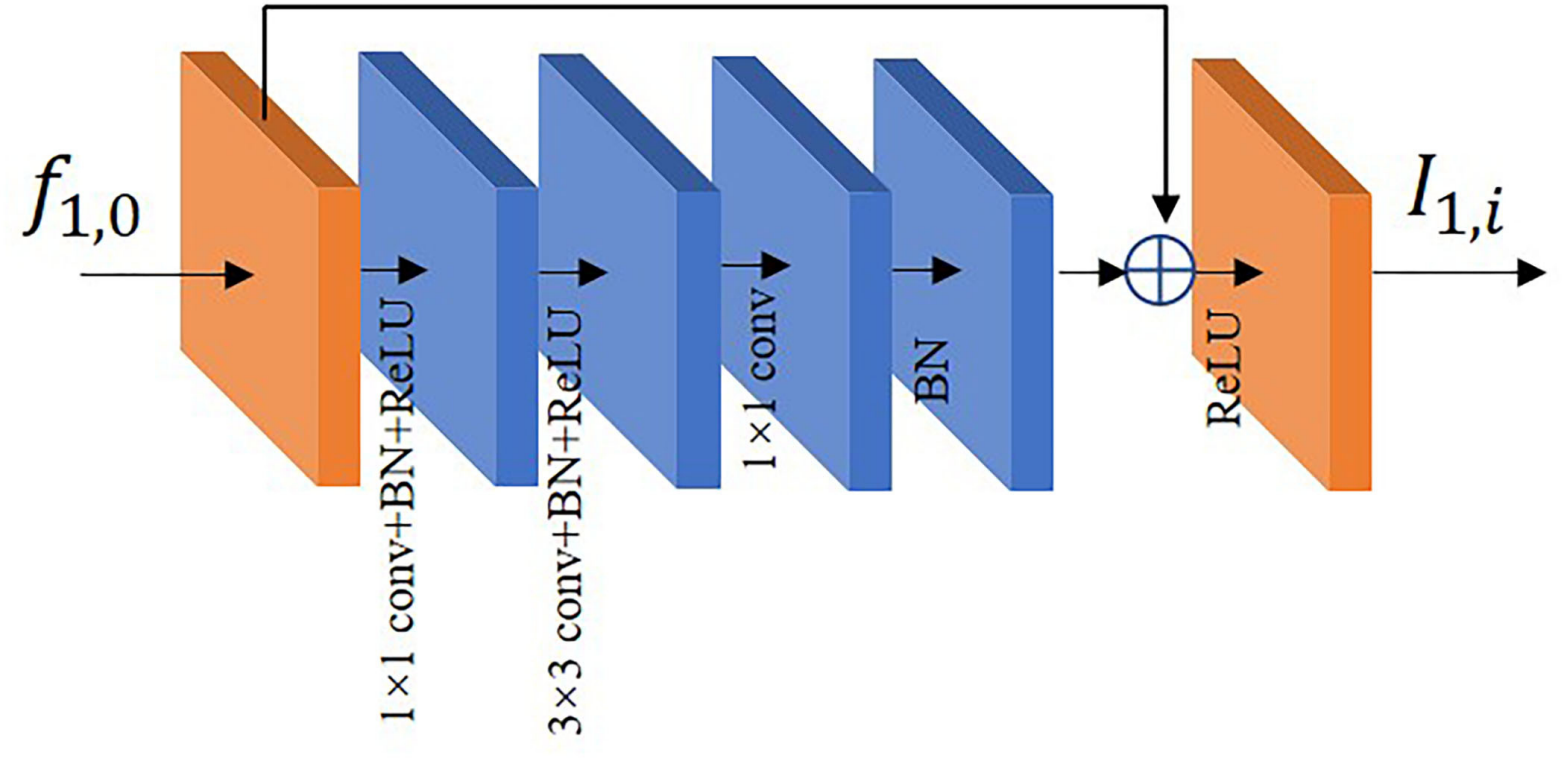
The residual block diagram.

Deeper networks are capable of extracting multi-level features (25, 26), especially in the high-level features where the feature information of target is more representative. In order to offset the performance degradation caused by network deepening (27), we use residual block (RB) in the path of low-resolution input to extract features. In the residual path, firstly, the dimensionality of the input features is reduced by 1 × 1 convolution. Then, 3 × 3 convolution is used to extract features in a larger receptive field. Finally, the features of each channel are fused by 1 × 1 convolution. The residual block utilizes an identity mapping approach. When the input features have reached a relatively optimal state, they are fed directly to the backend by identity mapping and the effect of residual path is adaptively weakened. The residual block used in this paper is shown in Figure 5. The formula for residual block is shown below:


(1) $$\begin{array}{l} I_{1,i}=ReLU\left(f_{1,0}+\delta \left(f_{1\times 1}\left(F_{3\times 3}\left(F_{1\times 1}\left(f_{1,0}\right)\right)\right)\right)\right) \end{array}$$

where *f*_1, 0_ and *I*_1, *i*_ denote the input and output of the residual block, respectively. *F*_*n*×*n*_ denotes *n*×*n* convolution, BatchNorm (BN) and ReLU activation function, *f*_*n*×*n*_ denotes *n*×*n* convolution, δ denotes the BatchNorm. The feature map *f*_1, 0_ of the previous output utilizes residual block to generate *I*_1, *i*_ which is used as part of the feature fusion.

### 2.4. Single dense aggregation block

**Figure 6:**
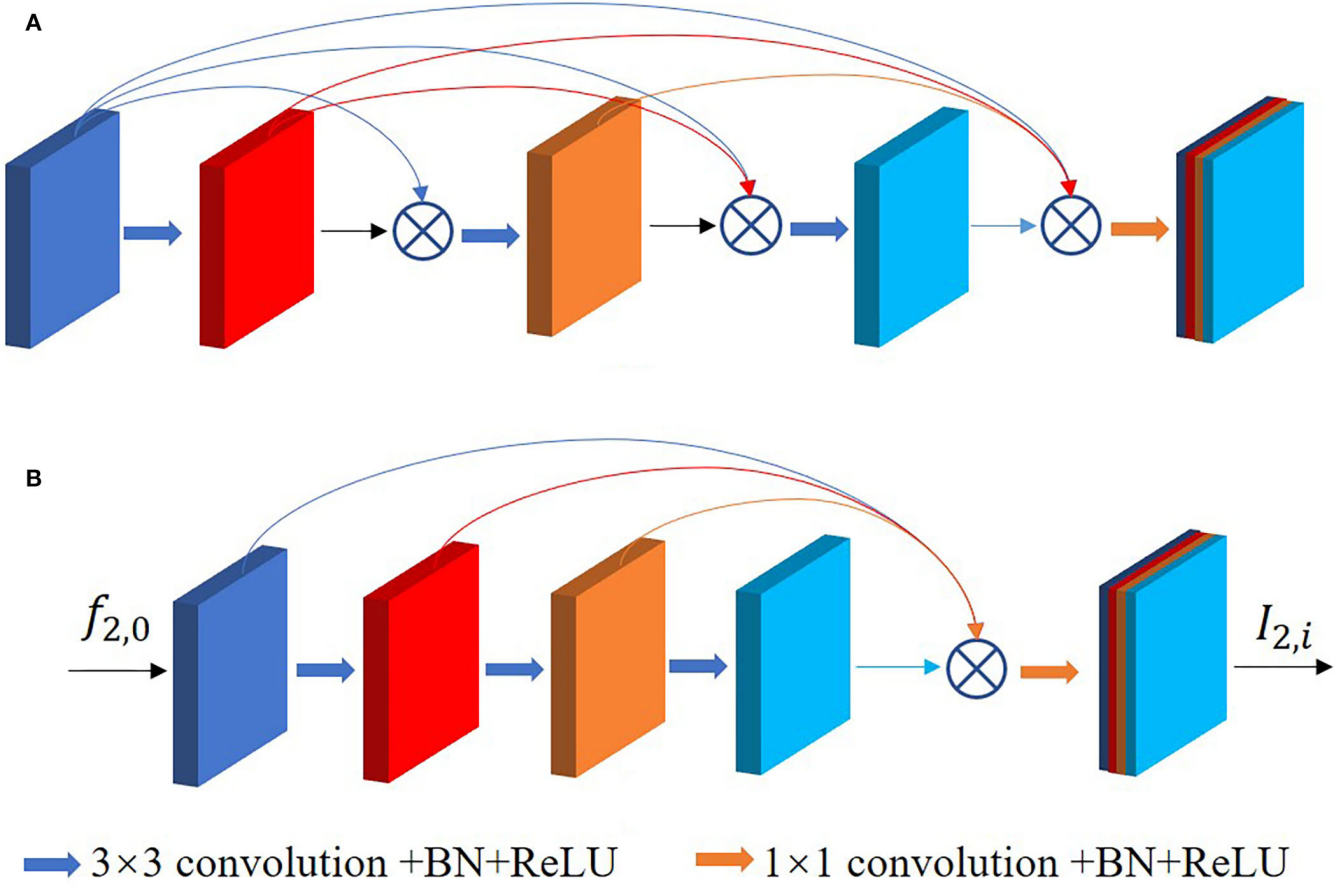
**(A)** Is the original dense connection, **(B)** is the single dense aggregation block used in this paper.

Dense connection learning and residual learning are two different methods to preserve and fuse features. The study by Huang et al. (28) shows the high correlation of the later layers with their neighboring layers and the low correlation with the more distant layers, which means that the deep features have low utilization of the shallow features. A large number of connections in the middle layers contribute little to the performance improvement and instead add significantly to memory redundancy, as shown in Figure 6A. Therefore, we adopt the single dense aggregation block (SDAB) (29) in the path of the high-resolution input as in Figure 6B. This method reduces the feature connections in the middle layers, and all the previous features are aggregated in the last layer by single connection, respectively. The formula for this block is expressed as follows:


(2) $$\begin{array}{l} f_{2,1}=\theta \left(W_{0}f_{2,0}\right)\\ f_{2,2}=\theta \left(W_{1}f_{2,1}\right)\\ \;\ldots \ldots \\ f_{2,l-1}=\theta \left(W_{l-2}f_{2,l-2}\right)\\ I_{2,i}=C\left(\left[f_{2,1},f_{2,2},\ldots ,f_{2,l-1}\right]\right) \end{array}$$

where *f*_2, 0_ and *I*_2, *i*_ denote the input and output of the single dense aggregation block, respectively, *f*_2, *j*_(1 ≤ *j* ≤ *l*−1) denotes the intermediate features, *W*_*j*_(0 ≤ *j* ≤ *l*−2) denotes the *j*^*th*^ layer 3 × 3 convolution, θ denotes ReLU activation function and BatchNorm, and *C*(^*^) denotes the connecting operation.

Since shallow features are involved in the final feature aggregation, this method can better retain the positional information and details of the tumor region, thus improving the efficiency of extracting features. The feature map *f*_2, 0_ of the previous output utilizes single dense aggregation block to generate *I*_2, *i*_ which is used as part of the feature fusion.

### 2.5. Multiscale feature fusion block

As stated in the introduction, some works on extracting features by multiple encoding paths have emerged to seek a relatively comprehensive aggregation of feature information of images. However, most of them ignore the connection between different encoding paths and do not effectively integrate the diversified semantic information in a multi-encoding feature flow.

**Figure 7:**
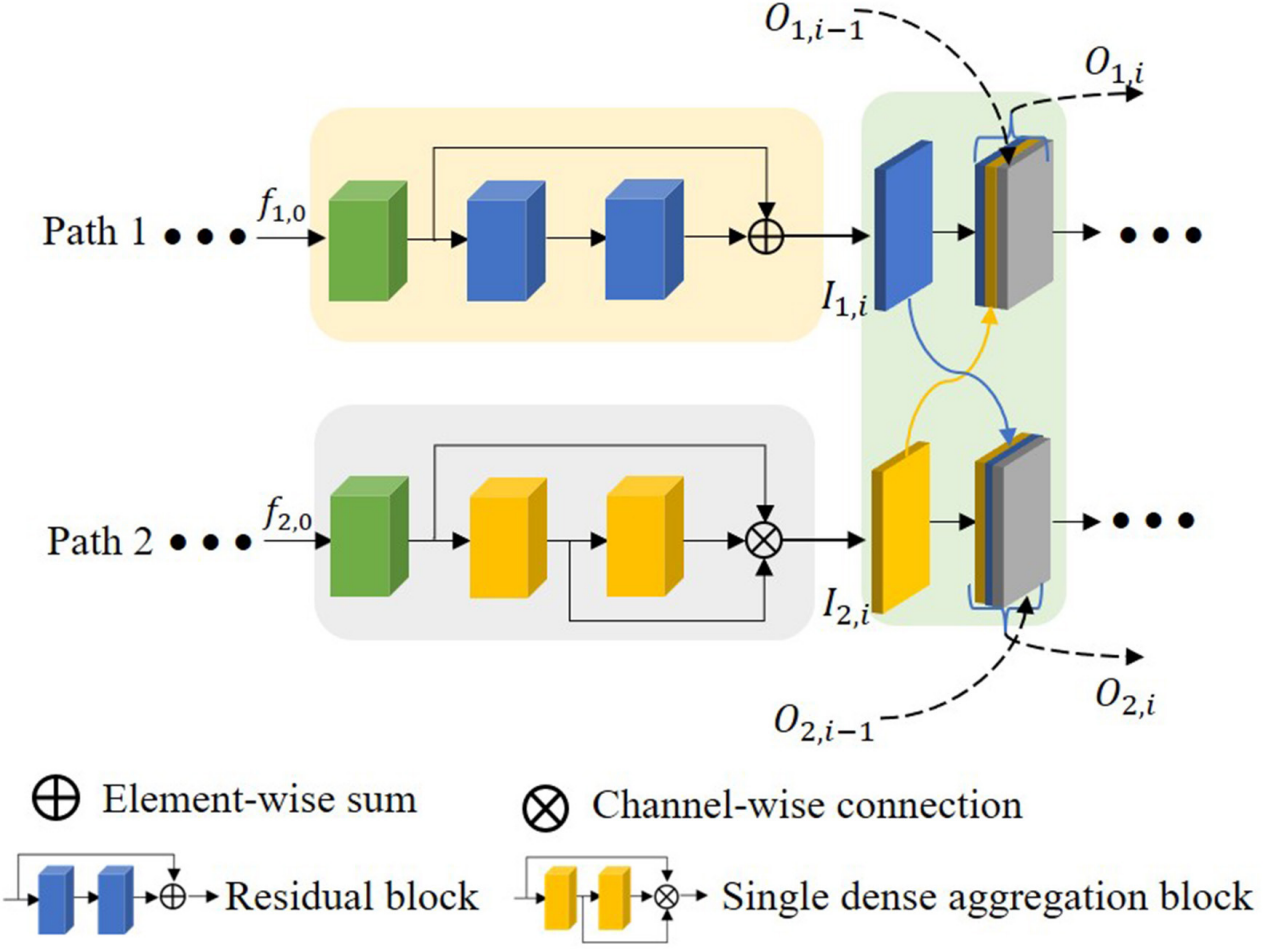
The proposed multiscale feature fusion block.

To respond to these issues, a multiscale feature fusion block (MFB) is proposed as shown in Figure 7. *O*_1, *i*_ denotes the output of the *i*^*th*^ block at path 1, *O*_2, *i*_ denotes the output of the *i*^*th*^ block at path 2, and the method of extracting features is different for each path. This block connects and fuses the multiscale features of each path in a different order, thus the proposed MFB enhances the correlation and diversity of features. In addition, the output of the previous layer is also directly involved in feature fusion in this layer to reduce as much feature loss in the deep network as possible. The first block does not have *O*_1, *i*−1_ and *O*_2, *i*−1_, and the fourth block does not have *O*_1, *i*_ and *O*_2, *i*_. The expression of the feature fusion connection is shown below:


(3) $$\begin{array}{l} O_{1,i}=C[I_{1,i},I_{2,i},O_{1,i-1}]\\ O_{2,i}=C[I_{2,i},I_{1,i},O_{2,i-1}] \end{array}$$

### 2.6. Coordinate attention

**Figure 8:**
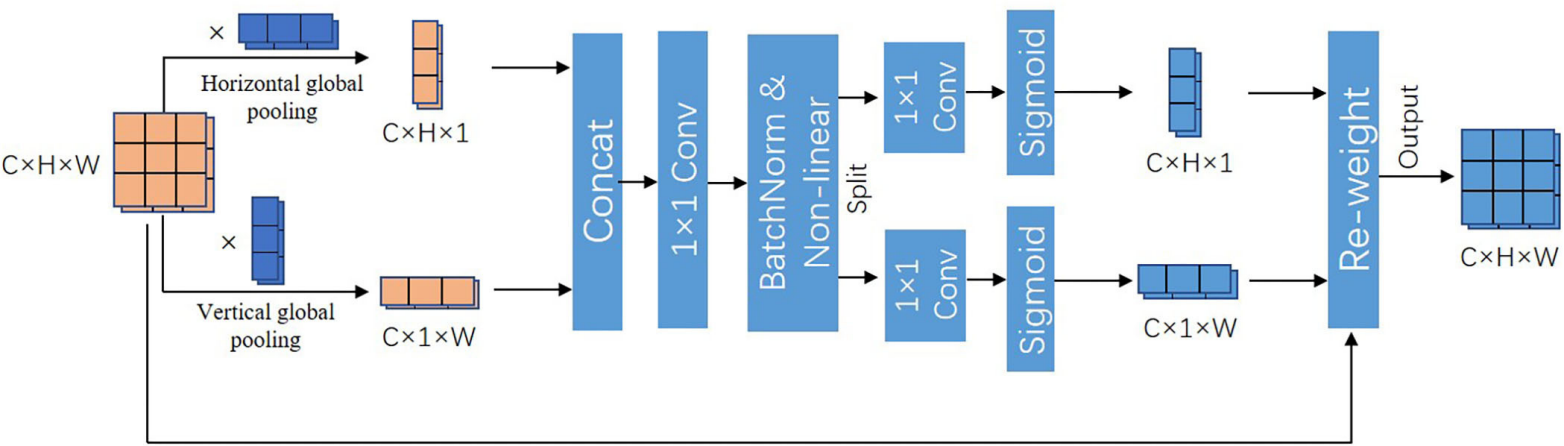
Coordinate attention.

Adding attention mechanism after concatenation layer can quickly filter out the valuable information and suppress the useless information. The coordinates attention (CA) diagram is shown in Figure 8. The global pooling is decomposed into two 1D encoders to facilitate the attention mechanism to be able to capture remote dependencies with precise positional information. Specifically, the input feature maps are pooled with pooling kernels of sizes (H, 1) and (1, W) performing average pooling for each channel in the transverse and longitudinal directions, respectively. The outputs of the features with width *w* and height *h* in the *c*^*th*^ channel are as follows:


(4) $$\begin{array}{l} z_{c}^{h}\left(h\right)=\frac{1}{W}\underset{0\leq i\leq W}{\sum }x_{c}\left(h,i\right) \end{array}$$

(5) $$\begin{array}{l} z_{c}^{w}\left(w\right)=\frac{1}{H}\underset{0\leq j\leq H}{\sum }x_{c}\left(j,w\right) \end{array}$$

We permuted the feature map of dimension C × 1 × W to C × W × 1, and concatenated it with the feature map of dimension C × H × 1 to obtain the feature map of dimension C × (H + W) × 1, and the result is encoded by performing 1 × 1 convolution, BatchNorm and non-linear function in turn. Then, the encoded feature maps are separated and then perform 1 × 1 convolution, followed by sigmoid activation function to generate the transversal and longitudinal attention. Finally, the obtained two attention maps are able to reflect whether the object of our interest exists in the corresponding rows and columns, which enables us to locate the target object accurately.

## 3. Experiments and results

### 3.1. Parameters setting

The research in this paper was done using PyTorch framework (30) and NVIDIA 3060Ti 8G GPU. The parameters were set as shown in Table 1. The batch size is 8 and iterations are 6,500. We used a loss function based on sample similarity, which is shown below:


(6) $$\begin{array}{l} loss=1-\frac{2\sum_{i}^{N}x_{i}y_{i}+1}{\sum_{i}^{N}x_{i}^{2}+\sum_{i}^{N}y_{i}^{2}+1} \end{array}$$

where *x*_*i*_ is the pixel-wise network prediction and *y*_*i*_ is the corresponding ground truth.

**Table 1:** Network parameters setting.

**Parameters**	**Values**
Optimizer	SGD
Initial learning rate	0.001
Momentum	0.9
Weight decay	0.0001
Learning rate change rule	$lr=lr\times {\left(1-\frac{iter\text{\_}num}{\max\text{\_}iter}\right)}^{0.9}$

### 3.2. Evaluation metrics

We utilized the dice similarity coefficient (DSC), Jaccard index (JI), sensitivity (Sen), specificity (Spe), and accuracy (Acc) to evaluate model performance. The evaluation results are obtained by comparing the network prediction with the ground truth pixel by pixel. The specific calculation of the metrics is shown below:


(7) $$\begin{array}{lll} DSC & = & \frac{2TP}{FN+FP+2TP} \end{array}$$

(8) $$\begin{array}{lll} JI & = & \frac{TP}{FP+FN+TP} \end{array}$$

(9) $$\begin{array}{lll} Sen & = & \frac{TP}{FN+TP} \end{array}$$

(10) $$\begin{array}{lll} Spe & = & \frac{TN}{TN+FP} \end{array}$$

(11) $$\begin{array}{lll} Acc & = & \frac{TP+TN}{TP+FN+FP+TN} \end{array}$$

where TP, FP, TN, and FN are true positive, false positive, true negative and false negative, respectively.

### 3.3. Quantitative analysis

To test the performance of DMFF-Net, we compared it with FCN (14), U-Net (15), DeepLabv3+ (31), Attention U-Net (32), STAN (21), and parallel deep learning algorithm (PDLA) (20), which are existing medical segmentation networks, and the comparison results are shown in Table 2. Table 2 shows that DMFF-Net achieved 91.6%, 84.8%, 92.5%, 99.5%, and 99.1% for DSC, JI, sensitivity, specificity, and accuracy, respectively, which outperforms the other networks in all metrics. We also counted the quantity of parameters for each network, where the least quantity of parameters is U-Net, but the DSC improved from 86.3% to 91.6%, while JI improved from 77.4% to 84.8%. Although the DSC of DMFF-Net is only 2.1% higher than that of STAN, its parameters are reduced by 42.05% compared to STAN. In addition, we used a paired-wilcoxon test on the DSC results to estimate the significant difference between the two models. When *P* < 0.05, it means that there are significant differences between the two models. It can be seen that DMFF-Net is significantly improved than other models. Compared with other networks, it can be concluded that the segmentation performance of the proposed network is significantly improved when the quantity of parameters is similar; the quantity of parameters of the proposed network is significantly reduced when the segmentation performance is similar.

**Table 2:** Quantitative comparison of DMFF-Net with other medical image segmentation networks.

**Methods**	**DSC (%)**	**JI (%)**	**Sen (%)**	**Spe (%)**	**Acc (%)**	**Para (M)**	**p-value**
FCN	69.3 ± 20.9	56.5 ± 21.4	80.4 ± 13.9	96.8 ± 3.5	95.9 ± 3.1	18.64	< 0.001 (***)
U-Net	86.3 ± 11.9	77.4 ± 15.1	84.8 ± 14.4	99.5 ± 0.4	98.8 ± 0.5	**7.85**	< 0.001 (***)
DeepLabv3+	86.5 ± 13.2	78.0 ± 14.6	88.2 ± 13.8	99.2 ± 0.5	98.7 ± 0.6	59.22	< 0.001 (***)
Attention U-Net	88.6 ± 8.4	80.5 ± 11.6	88.0 ± 11.1	99.5 ± 0.3	98.9 ± 0.4	34.87	< 0.05 (*)
STAN	89.5 ± 7.0	81.6 ± 9.7	90.2 ± 9.8	99.4 ± 0.3	98.9 ± 0.5	31.79	< 0.05 (*)
PDLA	90.6 ± 5.3	83.3 ± 8.1	92.0 ± 6.4	99.4 ± 0.3	99.0 ± 0.4	9.6	< 0.05 (*)
DMFF-Net	**91.6** **±** **4.9**	**84.8** **±** **7.6**	**92.5** **±** **7.1**	**99.5** **±** **0.2**	**99.1** **±** **0.3**	18.42	-

The bold values indicate the optimal result under the evaluation metrics.

^*^p < 0.05, ^**^p < 0.01, and ^***^p < 0.001.

**Figure 9:**
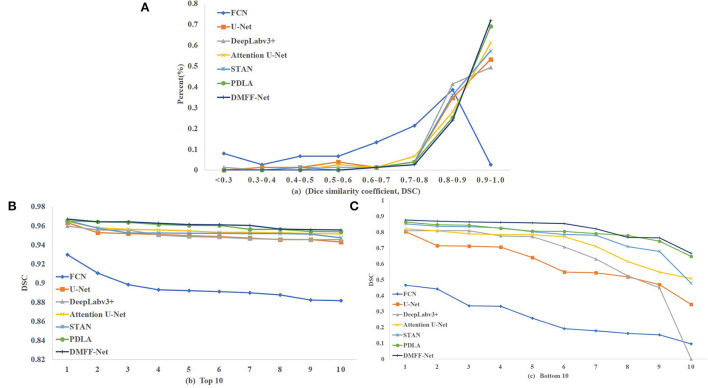
**(A)** Indicates the DSC distribution of test results for each model, **(B)** indicates the top 10 DSC for each model, and **(C)** indicates the bottom 10 DSC for each model.

We calculated the DSC of all the images in the test set, as shown in Figure 9. Figure 9A is the overall distribution of DSC, and it illustrates that DMFF-Net has the lowest percentage of DSC below 0.7 and the highest percentage above 0.9. Figures 9B, C are the results of the top 10 and bottom 10 of the DSC ranking, respectively. The segmentation results of DMFF-Net are basically at a high level for images that are easy to segment; for images that are difficult to segment, DMFF-Net has no cases of extremely poor segmentation or failure to segment. It is obvious that DMFF-Net has strong robustness for the segmentation of diverse ovarian tumor CT images.

### 3.4. Qualitative analysis

**Figure 10:**
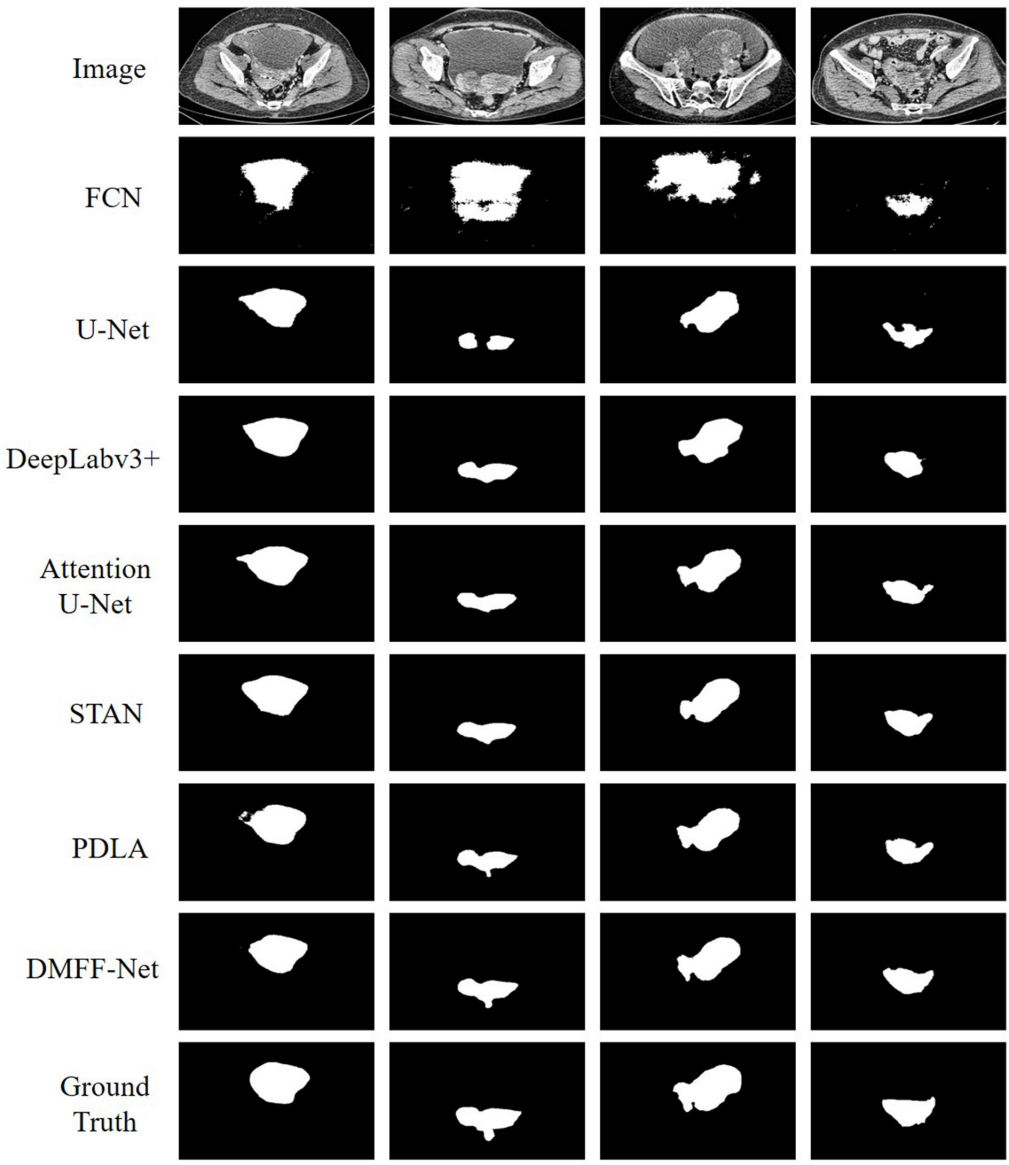
Qualitative comparison of segmentation results between DMFF-Net and other medical image segmentation networks.

To more visually represent the segmentation effect of different models on the ovarian tumors, we visualized the predictions and ground truth of the CT images participating in the test as shown in Figure 10. FCN has the coarsest segmentation and more false positive samples. U-Net and Attention U-Net can basically segment the lesion regions, which may be attributed to the shallow features providing the positional information of the target for the deep features through skip connections. But they both lack the segmentation of some details. STAN and PDLA can segment lesion details, but the results are not obvious. The proposed DMFF-Net can not only segment the lesion regions basically, but also outperforms other networks in detail segmentation. This is due to the fact that DMFF-Net performs the communication and fusion of multiscale features in the encoding stage, which makes the feature information more diverse.

### 3.5. Ablation experiment

#### 3.5.1. Ablation study for the quantity of convolution layers

In the process of designing the network structure, we explored the effect of setting the different number of 3 × 3 convolution layers in Figure 6B on the network performance, and the experimental results are shown in Table 3. The best performance is achieved when the quantity of convolution layers L is set to 10, and the worst performance is achieved when it is set to 4. This is because as the quantity of convolution layers increases, the higher the abstraction of the features, the more beneficial it is to segment the target accurately. In addition, the improved performance also brings an increase in the quantity of parameters.

**Table 3:** Effect of setting different convolution layers in single dense aggregation block on network performance.

**Layers**	**DSC (%)**	**JI (%)**	**Sen (%)**	**Spe (%)**	**Acc (%)**	**Para(M)**
L = 4	90.7 ± 7.2	83.7 ± 9.8	91.6 ± 9.0	99.5 ± 0.2	99.1 ± 0.5	**13.24**
L = 6	90.9 ± 6.7	83.9 ± 9.3	**92.6** **±** **6.7**	99.4 ± 0.4	99.0 ± 0.6	14.97
L = 8	91.4 ± 6.0	84.7 ± 8.8	92.4 ± 6.7	99.5 ± 0.2	99.1 ± 0.4	16.69
L = 10	**91.6** **±** **4.9**	**84.8** **±** **7.6**	92.5 ± 7.1	**99.5** **±** **0.2**	**99.1** **±** **0.3**	18.42

The bold values indicate the optimal result under the evaluation metrics.

#### 3.5.2. Ablation study for different encoding blocks

To research the impact of residual blocks and single dense aggregation blocks in DMFF-Net on the segmentation performance, we designed ablation experiment before and after module removal. The experimental results are shown in Table 4. When the residual blocks are removed from the encoding path, the DSC of DMFF-Net decreases from 91.6% to 90.2%, while the JI decreases from 84.8% to 83.1%; when the single dense aggregation blocks are removed from the encoding path, the DSC decreases from 91.6% to 85.6%. Thus, it can be seen that both the residual blocks and the single dense aggregation blocks contribute to the performance improvement of DMFF-Net. The contribution of the single dense aggregation block is larger.

**Table 4:** Effect of different encoding blocks in the dual encoding path on network performance.

**Methods**	**DSC (%)**	**JI (%)**	**Sen (%)**	**Spe (%)**	**Acc (%)**
Without RB	90.2 ± 8.8	83.1 ± 11.6	91.6 ± 10.9	99.5 ± 0.2	99.1 ± 0.5
Without SDAB	85.6 ± 11.7	76.3 ± 14.4	87.2 ± 12.1	99.2 ± 0.7	98.5 ± 0.8
DMFF-Net	**91.6** **±** **4.9**	**84.8** **±** **7.6**	**92.5** **±** **7.1**	**99.5** **±** **0.2**	**99.1** **±** **0.3**

The bold values indicate the optimal result under the evaluation metrics.

#### 3.5.3. Ablation study for multiscale feature fusion block and coordinate attention

The multiscale feature fusion block and coordinate attention utilized in this paper are two structures that barely increase parameters. To illustrate the effect of these two structures on the network performance, we removed them separately and experiment again. The experimental results are shown in Table 5. When the multiscale feature fusion blocks are removed, the DSC of the DMFF-Net decreases by 1.2%. In addition, the network performance also decreases when the coordinate attention is removed. From this experiment, it can be concluded that designing a high complexity model is not the only way to enhance the segmentation performance, and a reasonable structure can also contribute.

**Table 5:** Impact of multiscale feature fusion block and coordinate attention on network performance.

**Methods**	**DSC (%)**	**JI (%)**	**Sen (%)**	**Spe (%)**	**Acc (%)**
Without MFB	90.4 ± 10.3	83.7 ± 12.5	91.2 ± 11.9	99.5 ± 0.2	99.1 ± 0.5
Without CA	91.2 ± 5.6	84.4 ± 8.6	**94.5** **±** **5.4**	99.3 ± 0.3	99.1 ± 0.4
DMFF-Net	**91.6** **±** **4.9**	**84.8** **±** **7.6**	92.5 ± 7.1	**99.5** **±** **0.2**	**99.1** **±** **0.3**

The bold values indicate the optimal result under the evaluation metrics.

### 3.6. Experiment on the generalization ability of the model

To test the effectiveness of DMFF-Net for different segmentation tasks, we validated our model in the LITS 2017 (33), where we performed segmentation experiment on the liver. The dataset contains CT scans of the abdomen from 131 patients, we also performed a 5-fold cross-validation, and the experimental results are shown in Table 6.

**Table 6:** Quantitative comparison of liver segmentation results between DMFF-Net and other medical image segmentation networks.

**Methods**	**DSC (%)**	**JI (%)**	**Sen (%)**	**Spe (%)**	**Acc (%)**	**p-value**
FCN	90.0 ± 7.5	82.6 ± 10.2	94.6 ± 7.7	98.6 ± 1.9	98.4 ± 1.7	< 0.001 (***)
U-Net	90.7 ± 6.7	83.7 ± 9.5	**95.2** **±** **7.0**	98.7 ± 1.7	98.5 ± 1.5	< 0.001 (***)
DeepLabv3+	88.4 ± 7.2	79.8 ± 9.9	91.4 ± 8.7	98.9 ± 0.9	98.3 ± 0.9	< 0.001 (***)
Attention U-Net	90.6 ± 6.7	83.4 ± 9.5	95.0 ± 6.8	98.7 ± 1.8	98.5 ± 1.7	< 0.001 (***)
STAN	90.1 ± 6.8	82.5 ± 9.7	93.8 ± 7.4	98.7 ± 1.7	98.4 ± 1.5	< 0.001 (***)
PDLA	90.9 ± 6.8	83.9 ± 9.5	95.0 ± 7.0	98.8 ± 1.7	98.5 ± 1.6	< 0.01 (**)
DMFF-Net	**91.3** **±** **7.1**	**84.7** **±** **9.2**	94.6 ± 7.9	**99.0** **±** **1.2**	**98.7** **±** **1.2**	-

The bold values indicate the optimal result under the evaluation metrics.

^**^p < 0.01 and ^***^p < 0.001.

**Figure 11:**
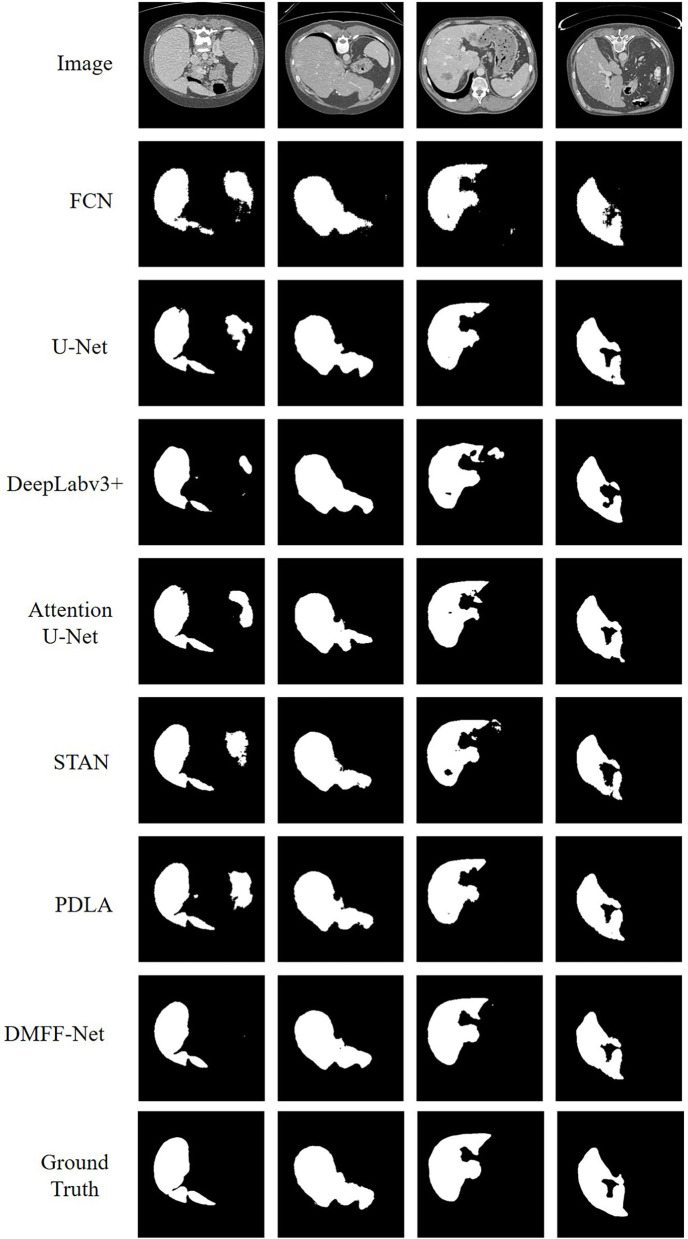
Qualitative comparison of liver segmentation results between DMFF-Net and other medical image segmentation networks.

**Figure 12:**
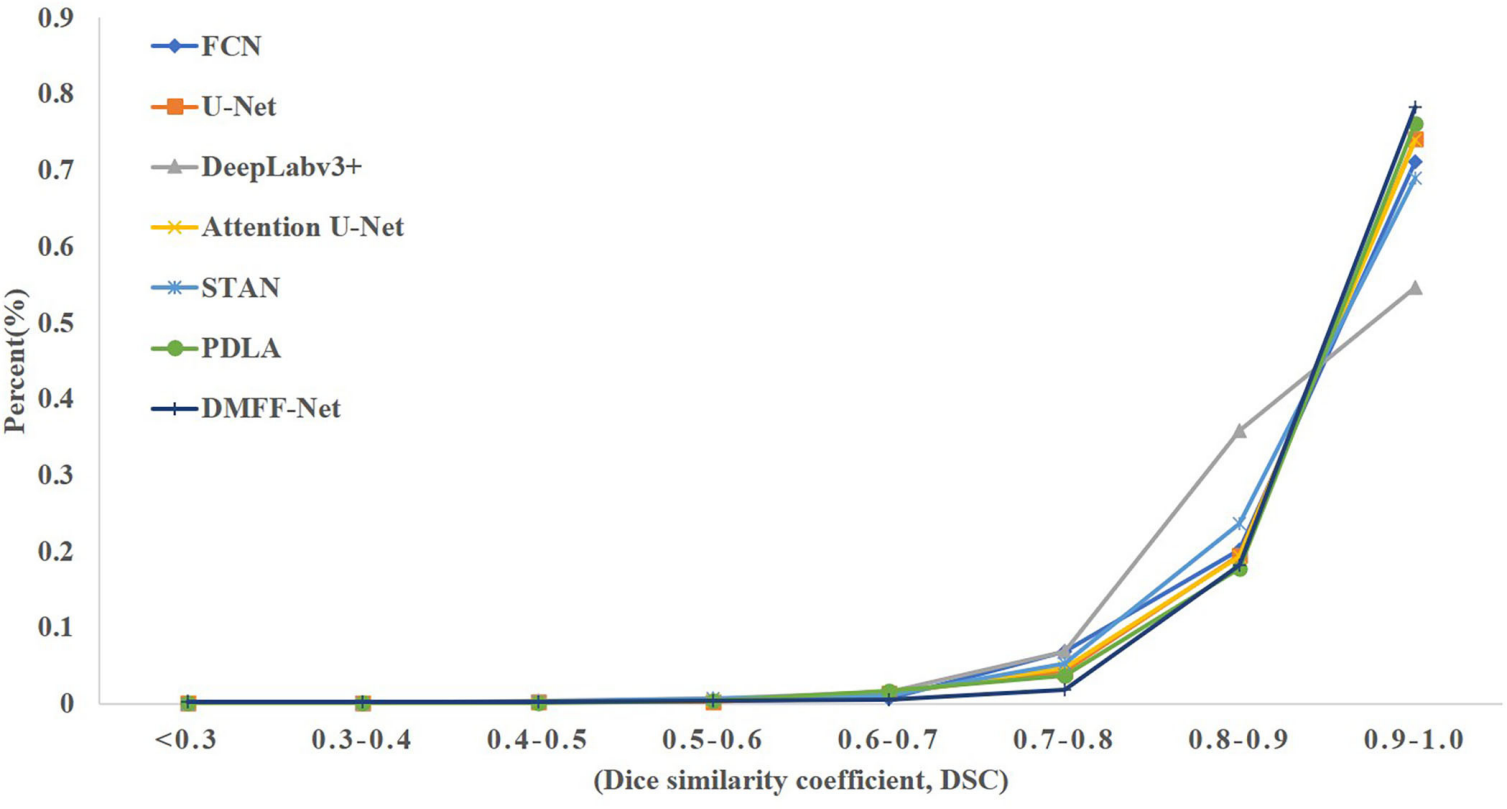
The DSC distribution on the liver test set for each model.

The proposed DMFF-Net outperforms other medical image segmentation networks in the liver segmentation task as well. DMFF-Net achieves a DSC of 91.3%, a JI of 84.7%, a Sen of 94.6%, a Spe of 99.0%, and an Acc of 98.7%. The visualized segmentation results are shown in Figure 11. Figure 11 shows that the segmentation of DMFF-Net for targets with large shape differences is closer to ground truth compared to other models and has fewer false positive pixels. Figure 12 shows the DSC distribution of different models on the liver segmentation test set, and the segmentation result of DMFF-Net has the highest percentage of DSC > 0.9.

Both the ovarian tumor and liver datasets are CT images, and the CT data are grayscale images with relatively single color. In order to verify the segmentation effect of the proposed method on non-CT data, we used the ISIC 2017 skin lesion dataset (34) to train and test the model, and the experimental results are shown in Table 7. The dataset was created for a skin damage analysis challenge called melanoma detection, where the number of images for training, validation and testing were 2,000, 150 and 600, respectively.

**Table 7:** Quantitative comparison of skin lesion segmentation results between DMFF-Net and other medical image segmentation networks.

**Methods**	**DSC (%)**	**JI (%)**	**Sen (%)**	**Spe (%)**	**Acc (%)**	**p-value**
FCN	76.2 ± 23.0	66.2 ± 24.7	73.7 ± 26.1	97.8 ± 4.7	90.4 ± 13.3	< 0.001 (***)
DeepLabv3+	75.1 ± 23.8	65.0 ± 25.7	78.5 ± 25.5	95.0 ± 13.2	89.5 ± 14.5	< 0.001 (***)
U-Net	78.6 ± 21.3	68.9 ± 23.7	76.7 ± 23.7	97.5 ± 5.6	91.2 ± 12.0	< 0.05 (*)
Attention U-Net	78.5 ± 21.6	68.8 ± 24.1	76.8 ± 23.9	97.4 ± 5.5	91.1 ± 12.3	< 0.05 (*)
STAN	79.3 ± 20.6	69.7 ± 23.3	76.5 ± 23.8	**97.9** **±** **4.7**	91.5 ± 11.9	< 0.05 (*)
PDLA	79.3 ± 20.6	69.6 ± 23.5	77.2 ± 23.4	97.7 ± 4.9	91.3 ± 12.4	< 0.05 (*)
DMFF-Net	**81.3** **±** **19.8**	**72.3** **±** **22.6**	**79.5** **±** **22.6**	97.5 ± 5.9	**92.0** **±** **11.8**	-

The bold values indicate the optimal result under the evaluation metrics.

^*^p < 0.05 and ^***^p < 0.001.

**Figure 13:**
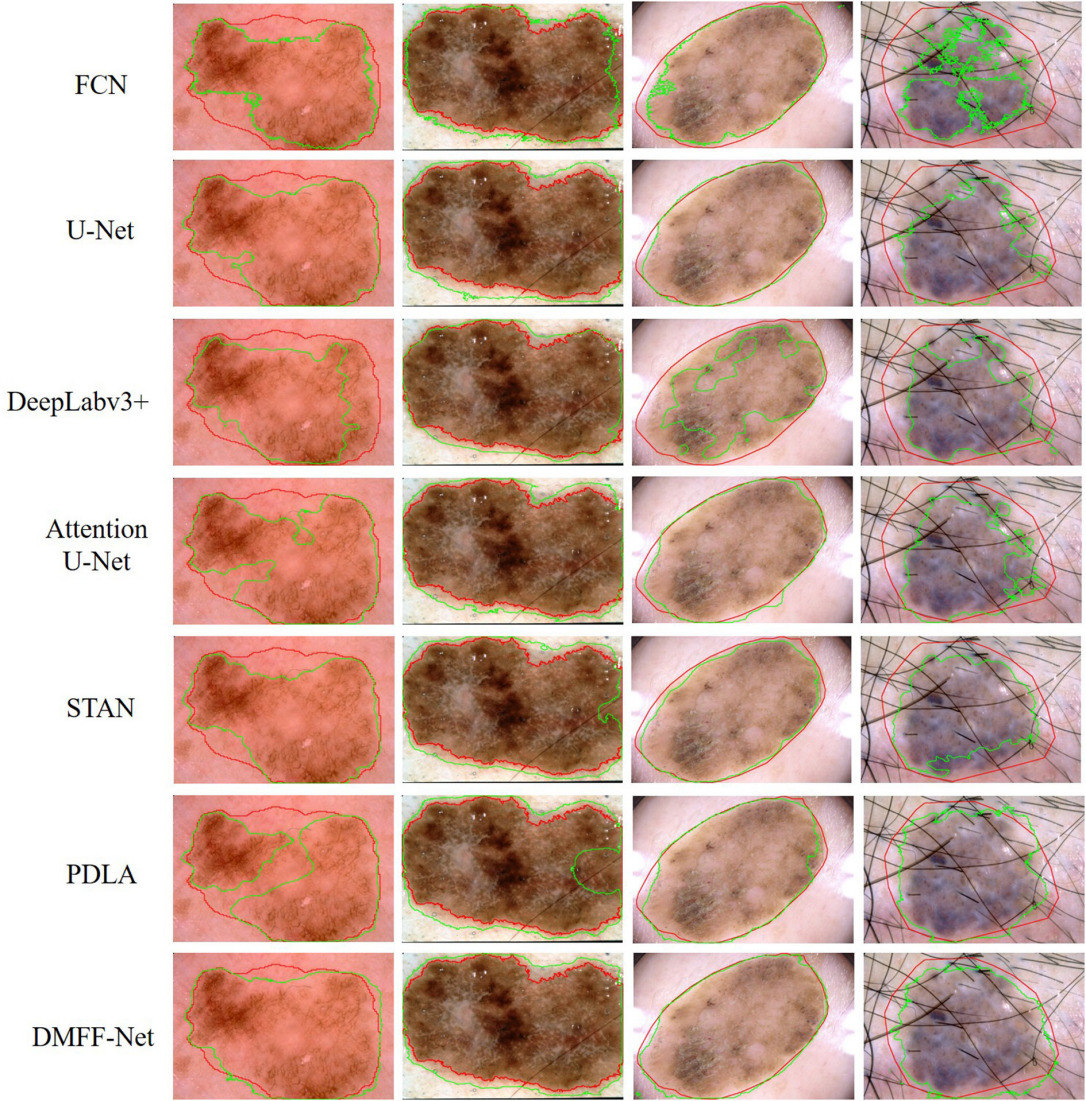
Qualitative comparison of DMFF-Net with other medical image segmentation networks for skin lesion segmentation results. The red line is ground truth and the green line is the prediction of the models.

**Figure 14:**
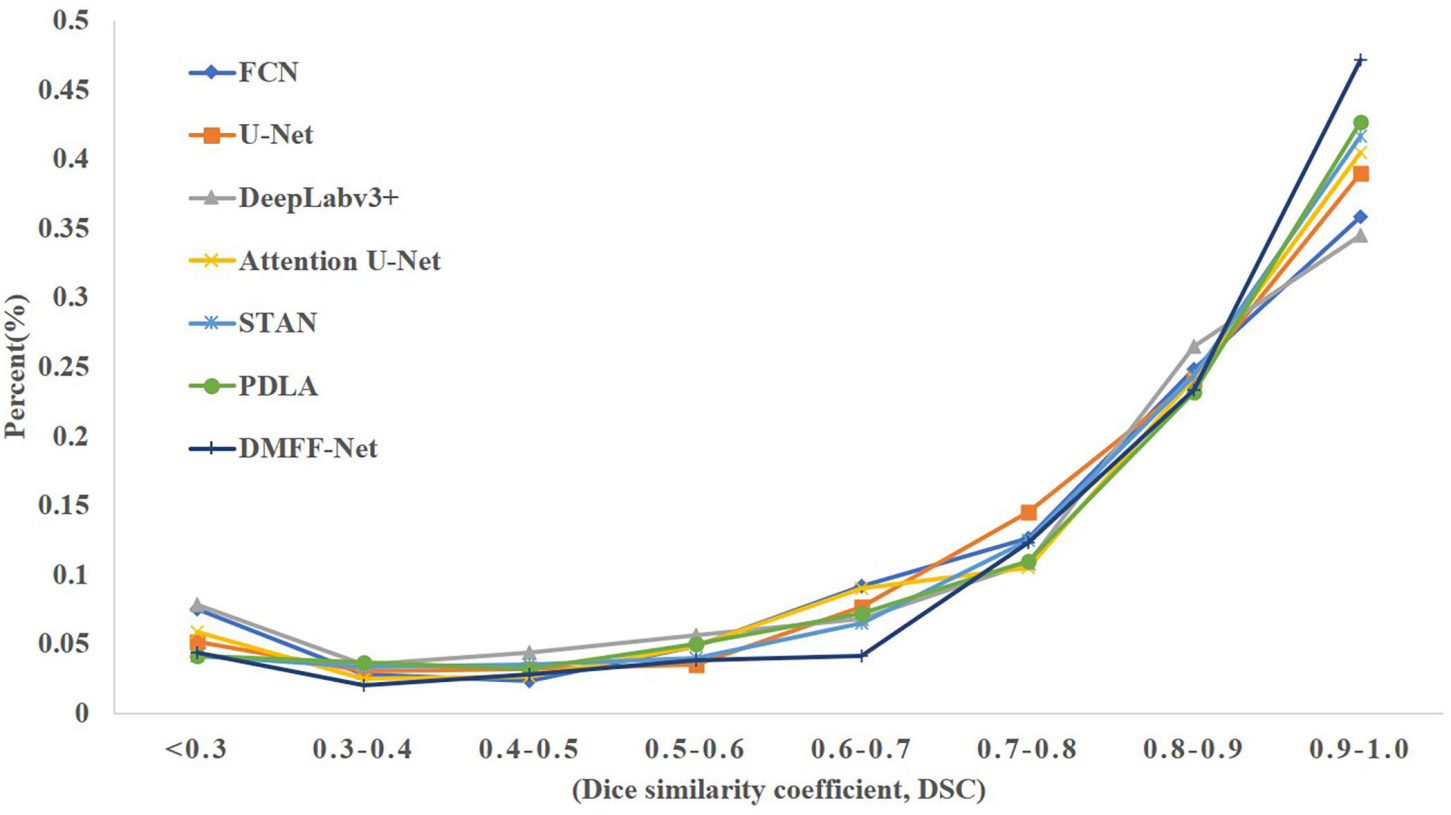
The DSC distribution on the skin lesion test set for each model.

Table 7 shows that DMFF-Net achieves the optimal segmentation effect compared with other models, and DSC is improved by at least 2.0%. Furthermore, compared to the performance on the other two datasets, the proposed method shows a greater improvement in segmenting skin lesions than the other networks. The visual segmentation results of the skin lesions are shown in Figure 13. We also compared with the latest works on the ISIC 2017 dataset and the results are shown in Table 8. It can be seen that the DSC of the DMFF-Net is 0.5% higher than the method proposed by Schlemper et al. (38), and the quantity of parameters is 40.93% of the latter. Figure 14 illustrates that DMFF-Net has a low percentage of DSC below 0.7 and the highest percentage above 0.9 in the test set.

**Table 8:** The comparison with the latest works on ISIC 2017.

**Methods**	**DSC (%)**	**JI (%)**	**Sen (%)**	**Spe (%)**	**Acc (%)**	**Para (M)**
Lin et al. (35)	79.0%	65.0%	-	-	-	-
Garcia-Arroyo and Garcia-Zapirain (36)	76.0%	66.5%	-	-	88.4%	-
Lin et al. (37)	77.2%	70.5%	**83.9%**	94.5%	91.8%	-
Schlemper et al. (38)	80.8%	-	79.9%	**97.7%**	91.4%	45 M
DMFF-Net	**81.3%**	**72.3%**	79.4%	97.5%	**92.0%**	**18.42 M**

The bold values indicate the optimal result under the evaluation metrics.

## 4. Conclusions

A dual encoding multiscale feature fusion network (DMFF-Net) for ovarian tumor segmentation is proposed in this paper. The network extracts diverse features by using different encoding structures. Multiscale feature fusion block explores the diversity between features of different paths to enrich feature information. In addition, the coordinate attention in the decoding stage can highlight the representation of effective information of the concatenated features. In the ovarian tumor segmentation task and two other segmentation tasks, the overall segmentation performance of the DMFF-Net outperforms other segmentation algorithms, and our segmentation is more accurate in details. Compared with other algorithms, the segmentation performance of the DMFF-Net is notably improved when the quantity of parameters is similar; the quantity of parameters of the DMFF-Net is notably reduced when the segmentation performance is similar. However, the segmentation accuracy still cannot fully meet the requirements of clinical diagnosis. In the next step, we will continue to investigate more efficient multiscale feature fusion methods.

## Data availability statement

Publicly available datasets were analyzed in this study. This data can be found at: https://challenge.isic-archive.com/data/#2017; https://academictorrents.com/details/~27772adef6f563a1ecc0ae19a528b956e6c803ce.

## Ethics statement

Written informed consent was obtained from the individual(s) for the publication of any potentially identifiable images or data included in this article.

## Author contributions

MW and GZ: conceptualization, writing—original draft, and methodology. MW and XW: writing—review and editing and funding acquisition. MW, GZ, XW, and ZW: validation. LW: resources. ZW: data curation. All authors contributed to the article and approved the submitted version.
